# Bridging TG‐116 and TG‐232: A retrospective multi‐vendor evaluation of deviation index re‐centering and process control in digital radiography

**DOI:** 10.1002/acm2.70820

**Published:** 2026-09-23

**Authors:** Ezzat AbuAzzah, Faisal Alrehily, Moawia Gameraddin, Sultan Alshoabi, Walaa Alsharif, Kamal Dahan Alsultan, Abdullah Alshamrani, Khaled Al‐Radadi, Ramy Badawy, Samer Al‐Jabri, Turki Al‐Harbi, Nawaf Al‐Hazme

**Affiliations:** ^1^ Department of Diagnostic Radiology College of Applied Medical Sciences Taibah University Madinah, Al Madinah Province Kingdom of Saudi Arabia; ^2^ Medical Physics Department King Fahd Hospital Madinah, Al Madinah Province Kingdom of Saudi Arabia

**Keywords:** deviation index, digital radiography, exposure index, machine learning, QA triage, quality assurance, target exposure index, TG‐116, TG‐232

## Abstract

**Background:**

The deviation index (DI) is a vendor‐neutral indicator of detector exposure relative to the configured target exposure index (TEI). Because TEI is a protocol value rather than a calibrated physical quantity, poorly aligned TEI settings can create systematic DI offsets, obscure protocol problems, and prevent meaningful statistical process control.

**Objective:**

This retrospective multi‐vendor study evaluated whether routine DICOM metadata can identify biased DI distributions, separate fixed TEI‐setting offsets from protocol‐adherence variation, estimate candidate TEI updates, and support TG‐232‐aligned standard deviation‐based monitoring after DI re‐centering.

**Methods:**

We retrospectively analyzed 38 916 digital radiography examinations performed from January 1, 2024 through August 27, 2024 using three vendor platforms at a tertiary hospital. DICOM metadata were benchmarked against published technical standards using a composite adherence index (CAI) for SID, kVp, mAs, grid use, and AEC use. A secondary gradient‐boosting/SHAP analysis was retained as a non‐causal variance discovery to rank the metadata patterns used to predict DI. Candidate TEI updates were estimated within the vendor‐region‐projection groups by algebraically re‐centering the observed mean DI ($\bar{DI})$ to 0.0. Robustness was assessed using sensitivity analyses restricted by automatic exposure control, beam energy, and patient habitus proxy measures.

**Results:**

The baseline $\bar{DI}$ = −3.1 (SD 3.7), and only 11.3% of examinations were within |DI| ≤ 1. Forty of 45 vendor–region–projection groups (88.9%) had an absolute group $\bar{DI}$ greater than 1.0. Separately, the mean CAI was 0.176. The secondary SHAP analysis ranked the configured TEI, manufacturer, SID, exposure, and kVp as high‐contribution predictors of DI; these findings were interpreted as predictive attribution, not causal evidence. DI re‐centering reduced the overall variance by 42.9% (13.5 to 7.7), increased the proportion of examinations within |DI| ≤ 1 to 34.3%, and reduced severe deviations (|DI| > 3) from 60.3% to 23.1%.

**Conclusion:**

In this retrospective, multi‐vendor study, the TEI setting review was operationalized as a structured, data‐driven responsibility of a clinical site. By verifying protocol adherence, equipment performance, image quality, and dose indicators before centering the $\bar{DI}$, departments can support the conversion of the DI from a biased compliance signal into a practical process‐control metric for exposure governance.

## 1 INTRODUCTION

The transition to digital radiography (DR) has fundamentally altered the relationship between radiation dose and image quality. Unlike screen‐film systems, in which overexposure results in diagnostic failure, digital detectors possess a wide dynamic range that allows the production of diagnostically acceptable images even at substantially elevated dose levels.^1^ This decoupling of acquisition and display has introduced the risk of dose creep, in which exposure factors are incrementally increased without immediate feedback to correct the trend.^2^ To address this, the International Electrotechnical Commission (IEC) and the American Association of Physicists in Medicine (AAPM) Task Group 116 (TG‐116) established the deviation index (DI) as a standardized, vendor‐neutral exposure feedback metric.^3^, ^4^ DI is defined as:

(1) $$DI=10\times \log_{10}\left(\frac{EI}{TEI}\right)$$

where EI is the exposure index reported by the DR system, and TEI is the configured target exposure index (TEI) for the examination. In the IEC 62494‐1 and TG‐116 terminologies, EI is related to the detector air kerma under specified calibration conditions,^3^, ^4^ whereas TEI is a protocol target selected by the site/vendor implementation. A DI of 0.0 indicates that the reported EI matches the configured targets.

However, the utility of the DI depends on whether the configured TEI represents the detector exposure needed for the local protocol, image quality expectation, and vendor implementation. EI and AEC systems are calibrated performance quantities, but TEI is a configurable protocol value. In practice, TEI values may remain at vendor defaults or historical local settings that do not reflect local preferences, beam qualities, detector behavior, AEC thresholds, or workflow needs.^5^, ^6^, ^7^, ^8^ This creates a governance gap: DI distributions become systematically offset from zero, making it difficult to distinguish technologist variability, AEC performance problems, detector/EI calibration issues, and poorly aligned TEI settings.^7^, ^8^, ^9^ Although AAPM TG‐232 recommends site‐specific standard deviation (SD)‐based control limits for DI interpretation,^10^ such limits are meaningful only after the mean DI ($\bar{DI})$ has been centered around an appropriate target.

Persistent TEI misalignment has direct consequences on patient safety and quality. If the configured TEI is too low, the DI may suggest overexposure and encourage unnecessary dose reductions. If the configured TEI is too high, appropriate exposures may be labelled underexposed, encouraging dose creep.^2^ Chronic offset also promotes alarm fatigue; when the DI is persistently non‐zero because the configured target is poorly aligned, genuine outliers are masked by false positives.^10^, ^11^ Without a credible centered baseline, the TG‐232‐style process control cannot be implemented.

This retrospective multi‐vendor study evaluated a site‐governed TEI‐setting review framework. Rather than treating TEI as a static vendor default, we tested whether routine DICOM metadata could (a) screen protocol adherence using a composite adherence index (CAI), (b) review DI behavior explicitly within vendor‐region‐projection groups, (c) use machine learning as a secondary, non‐causal variance‐discovery layer to rank which metadata patterns a predictive model uses when estimating DI, and (d) estimate candidate TEI settings by algebraically re‐centering group‐level $\bar{DI}$, thereby enabling the SD‐based control limits recommended by TG‐232.^10^

## 2 MATERIALS AND METHODS

### 2.1 Study design and setting

We retrospectively analyzed 38 916 consecutive DR examinations performed from January 1, 2024, to August 27, 2024, at a tertiary facility using Samsung (DGR‐C55J29/WR), GE (Discovery XR656), and Carestream (DRX‐Evolution) imaging systems. The study was approved by the relevant institutional review board (approval no. 25‐003), and all data were anonymized before analysis.

The analytic dataset included consecutive DR records with non‐missing manufacturer, model, sex, series date, body part, body region, projection/view, SID, AEC mode, EI, TEI, DI, kVp, exposure, age group, and grid‐use fields. These key fields were complete for all 38 916 exported records; therefore, no examination was excluded for missing key metadata after anonymized PACS export. Because anonymization removed patient identifiers and repeat/reject labels, repeat acquisitions could not be identified reliably. They were intentionally retained as acquisition‐level operational QA observations so that technique‐related outliers remained visible; however, repeats may widen the CAI or DI distributions, and the results should not be interpreted as patient‐level estimates.

The responsibilities for the TEI configuration at the study site were implicitly divided. Detector acceptance testing and major configuration changes were performed by vendor service teams, and day‐to‐day protocol work was performed by technologists under the supervision of medical physicists. Routine TEI review, DI statistics monitoring, comparison of configured TEI with clinically observed EI/DI behavior, and controlled TEI‐table updates were neither specified in vendor service contracts nor included in the internal QA schedule.^9^ Consequently, no standardized mechanism existed for either the vendor or the clinical team to identify poorly aligned TEI settings and initiate a review. Staff observed that DI values were chronically far from zero and clustered asymmetrically but lacked a formal process to determine whether this reflected TEI bias, protocol noncompliance, AEC behavior, detector/EI calibration, or patient‐mix variation. This operational gap motivated the workflow described in this study.

### 2.2 Data source and variables

All examinations were extracted from the PACS archive using in‐house software capable of searching and retrieving DICOM metadata. The primary endpoint was the examination‐level DI reported at each system's native vendor precision; DI was therefore treated as a quantized rather than truly continuous value (single precision 0.1 increments in all three systems used by this study). The derived operational measures included examination‐level CAI, examination‐level DI‐band proportions, group‐level mean $\bar{DI}$ and $|\bar{DI}|$, group‐level TEI‐review flags, and counterfactual post‐re‐centering DI distributions. The arithmetic $\bar{DI}$ was selected because the algebraic re‐centering transformation and variance/SD‐based process control target the group mean to maintain additive accuracy on the logarithmic scale, where $\bar{DI}$ corresponds to the geometric mean EI‐to‐TEI ratio. Median and percentile summaries remain useful robust complements for skewed groups, but they were not used to calculate candidate TEIs. The predictor variables were organized into three groups:

- The technical parameters included the EI, TEI, tube potential (kVp), exposure (mAs), source‐to‐image distance (SID), grid use, and automatic exposure control (AEC) mode.^12^, ^13^
- The protocol descriptors included body region, projection view, and age grouping mapped to consolidated categories reflecting institutional protocols.
- The contextual factors included the manufacturer and patient sex.

The technical parameters were benchmarked against the AAPM TG‐150^14^ and ASRT best practices^15^ using region‐ and view‐specific target ranges and tolerances for SID, kVp, mAs, grid use, and AEC. TEI‐setting alignment was assessed using the group‐level mean $|\bar{DI}|$ within each vendor‐region‐projection group. A sustained $|\bar{DI}|$ above 1.0 was interpreted as evidence of a fixed group‐level offset requiring review, not as proof that the TEI alone was wrong.

Vendor‐region‐projection groups were defined by the manufacturer, consolidated body region category, and projection/view label. All 45 observed groups were retained for descriptive TEI‐review screening, with group sizes ranging from 2 to 5328 examinations. Groups with very small counts were interpreted as screening signals requiring local review rather than as standalone evidence for immediate TEI‐table changes.

### 2.3 Statistical analysis

For each examination, we derived deviation metrics that described both protocol adherence and TEI‐setting alignment. These included absolute and percentage deviations of the SID, kVp, mAs, grid use, and AEC relative to region‐ and view‐specific standards, binary out‐of‐spec flags, a CAI, and group‐level TEI‐review flags based on the $|\bar{DI}|$.

The statistical plan followed an endpoint hierarchy. Descriptive summaries characterized examination‐level DI and CAI; vendor‐region‐projection $|\bar{DI}|$ flagged groups for TEI‐setting review; counterfactual re‐centering assessed variance and DI‐band proportions at the examination level after group‐level mean subtraction; Welch tests, Welch ANOVA, two‐way ANOVA, bootstrap confidence intervals, and Pearson correlations were reserved for sensitivity analyses; and ML/SHAP was treated as secondary exploratory QA triage.

To summarize the acquisition quality independently of the detector calibration, we defined the CAI for examination i as:

(2) $${\mathrm{CAI}}_{i}=\frac{1}{N}\;\sum_{j=1}^{N}\left(1-C_{\mathrm{ij}}\right)$$

where N=5 is the number of technical domains evaluated, and $C_{ij}$ is a binary compliance flag for parameter j in examination *i* (1 = compliant, 0 = non‐compliant). Thus, CAI=0.0 indicates perfect adherence, whereas larger values indicate progressively greater protocol deviations.

Compliance was evaluated against region‐ and age‐specific standards derived from the AAPM TG‐150 and ASRT best practices. The SID was considered compliant if it was within ± 2% of the prescribed distance (100 or 180 cm, depending on anatomy). The kVp and mAs values were considered compliant if they fell within the anatomy‐specific reference ranges with tolerances of ± 5% and ± 10%, respectively. The ± 5% kVp and ± 10% mAs bands were deliberately sensitive metadata‐audit bands around protocol quality control (QC) references, not universal clinical acceptance limits. Patient thickness, pathology, positioning, and AEC selection may legitimately change mAs by more than 10%; therefore, a flagged value contributed to CAI only as a prompt for QC contextual review and was not treated as proof of an acquisition error. The grid was mandatory for adult torso and spine examinations and optional for pediatric and extremity examinations. An AEC was recommended for adult torso imaging but was optional for other regions.

Head examinations labelled as cervical spine or neck studies in the DICOM body part field were evaluated against spine rules, and the remaining head examinations were evaluated against skull‐specific rules. This allowed the standards‐based CAI computation to be applied consistently to the entire cohort.

This formulation allowed us to separate procedural non‐compliance from the fixed DI offset. A vendor‐region‐projection group with an elevated $\bar{DI}$ but low mean CAI suggests a TEI‐setting or equipment‐performance issue, whereas a concurrent elevation of DI and CAI suggests broader protocol variation.

### 2.4 Secondary machine‐learning QA triage

A secondary machine‐learning analysis was applied as a variance‐discovery and QA‐triage step, not as the primary method for deriving TEI values, and not as evidence of causality. A prespecified gradient boosting model^16^ was trained to predict the DI from DICOM‐derived technical variables, protocol descriptors, and contextual factors. Categorical variables were one‐hot encoded, continuous variables were retained on their natural scales, and the dataset was split into an 80% training set (*n* = 31 132) and a 20% held‐out evaluation set (*n* = 7784), stratified by body region. The retained model used 200 estimators, a learning rate of 0.05, a maximum depth of 7, a minimum sample split of 2, a subsample of 0.8, and a random state of 42 after hyperparameter optimization. Hyperparameters for the retained gradient‐boosting model were selected using a randomized search over a predefined grid implemented with scikit‐learn's RandomizedSearchCV (fixed random state 42). All random procedures used a fixed seed 42.

To keep the main research article focused on the DI/TEI‐setting analysis, comparative model performance results were not retained as a main‐text table. The selected model was used for SHapley Additive exPlanations (SHAP)‐based triage only after the held‐out evaluation confirmed a useful predictive structure for ranking metadata patterns; RMSE and MAE are reported transparently in the Results rather than being used as evidence of causality.

The selected model was then analyzed with SHAP.^17^ SHAP assigns a contribution score to each feature for each prediction, allowing us to identify the metadata patterns on which the predictive model relied when estimating the DI. SHAP values were computed on a random sample of 2000 examinations from the held‐out set using random state 42, and the global rankings were based on the mean absolute SHAP values. Because DI is algebraically linked to EI and TEI, TEI importance was expected; it was interpreted as a QA signal for configured‐target reviews rather than a causal claim. The model did not derive updated TEI values and was not used to replace CAI, clinical image quality review, dose review, detector EI verification, or AEC performance testing.

### 2.5 TEI re‐centering simulation

To estimate the effect of removing fixed group‐level DI offsets, we used an algebraic TEI re‐centering simulation derived from the definition of DI. Rearranging Equation 1, the examination‐level target that would center a given DI value to zero can be written in plain text as $TEI_{center,i}=EI_{i}\times 10^{-\frac{DI_{i}}{10}}$. At the examination level, this implied that the TEI should equal the configured TEI when the DICOM metadata were internally consistent; we used this identity as a metadata quality screen before proceeding. At the vendor‐region‐projection group level, substituting the group mean for the deviation index $DI_{s}$ and the currently configured exposure target $TEI_{s}$ yield the candidate proposed target $TEI_{new,s}$ shown in Equation 3.

(3) $$TE\;I_{new,s}=\mathrm{TE}I_{s}\;\times 10^{DI_{s}/10\;}$$

A positive group mean therefore requires a higher candidate TEI. Equivalently, $TEI_{new,s}$ equals the geometric mean $EI_{s}$ of examination‐level EI values within the group, providing an intuitive link between the re‐centering target and the observed detector exposure.

The TEI‐setting review was conducted at the vendor‐region‐projection group level. We performed three steps for each group. First, we verified that the examination‐level implied TEI values matched the configured TEI values as a metadata consistency screen. Second, we estimated the fixed DI offset as the $\bar{DI}$ of the group. Third, we constructed a counterfactual DI distribution by subtracting that group‐specific mean from each observation DI_i_ in the same group.

(4) $$DI_{i}^{cf}=DI_{i}-DI_{s}$$

This operation shifts the group mean but does not remove within‐group variance; observations remain spread around the new center according to their original technique, patient, AEC, detector, positioning, and protocol variations.

We quantified the achievable improvement using the variance reduction (VR) metric defined as

(5) $$VR\left(\%\right)=\frac{\sigma^{2}\left(DI\right)-\sigma^{2}\left(DI^{cf}\right)}{\sigma^{2}\left(DI\right)}\;\times 100\%$$

In Equation 5, $\sigma^{2}\left(.\right)$ stands for variance. This metric estimates the proportion of the total DI variance removed by eliminating fixed group‐level offsets. This does not imply that the remaining within‐group variance, image quality, or patient dose has been optimized.

### 2.6 Sensitivity analyses

To test robustness against plausible confounders, we performed three prespecified sensitivity analyses. First, we restricted the analysis to AEC‐only examinations to reduce confounding from manual exposure selection.^18^ Second, because beam energy influences the detector response and patient penetration, we stratified the cohort into four diagnostic kVp bands (≤ 70, 71–90, 91–110, and > 110 kVp) corresponding to the clinical ranges for extremity, chest, abdomen, and high‐kVp chest imaging. Welch ANOVA was used rather than standard ANOVA because vendor groups had unequal variances and sample sizes. Pairwise comparisons were used as exploratory robustness checks to test whether vendor‐specific DI offsets persisted within narrow beam energy ranges. Third, because direct BMI data were unavailable, we used log10(1 + mAs) as a proxy for patient habitus,^8^ because mAs increases with patient attenuation under AEC and therefore reflects body thickness. We calculated Pearson correlations between the DI and this proxy within each sensitivity subset.

Because AEC uptake was strongly vendor‐correlated (99.9% Carestream, 24.5% Samsung, and 14.6% GE AEC rates), the AEC‐only subset could not serve as a vendor‐neutral control. To address this imbalance, we additionally: (i) tabulated vendor × AEC‐mode cell counts and per‐cell $\bar{DI}$ and SD to expose the composition bias; (ii) for vendors with both AEC and MANUAL arms, performed within‐vendor Welch t‐tests on DI and bootstrapped the mean difference using 1000 examination‐level resamples to produce percentile 95% confidence intervals for the AEC‐versus‐manual effect within vendor; and (iii) fitted a two‐way ANOVA on DI with vendor, AEC‐mode, and their interaction as fixed effects on the balanced two‐vendor subset (Samsung Electronics, GE Healthcare), because Carestream Health contributed no MANUAL examinations and could not be included in the factorial design. For each effect we report partial eta‐squared $\eta_{p}^{2}$ and, for completeness, total eta‐squared $\eta^{2}$. For one‐way Welch ANOVAs by kVp band, $\eta_{p}^{2}$ equals $\eta^{2}$ and both are reported. The two‐way ANOVA thus yields distinct values for vendor, AEC‐mode, and their interaction.

### 2.7 Scientific computing environment

All analyses and visualizations were performed using Python 3.9.^19^ Data manipulation was performed using NumPy and pandas,^20^, ^21^ statistical testing using SciPy,^22^ secondary predictive modeling using scikit‐learn,^23^ model interpretability using SHAP,^17^ and figures were generated using Matplotlib and Seaborn.^24^, ^25^ In addition to conventional scientific computing tools, Claude and Gemini were used to support parts of the analytical exploration and interpretation workflows. All computational outputs were reviewed and verified by the authors, and the final codebase was executed on a standard workstation. The code is available from the corresponding author upon reasonable request.

## 3 RESULTS

### 3.1 Baseline DI behavior, protocol deviations, and TEI‐setting review

**FIGURE 1:**
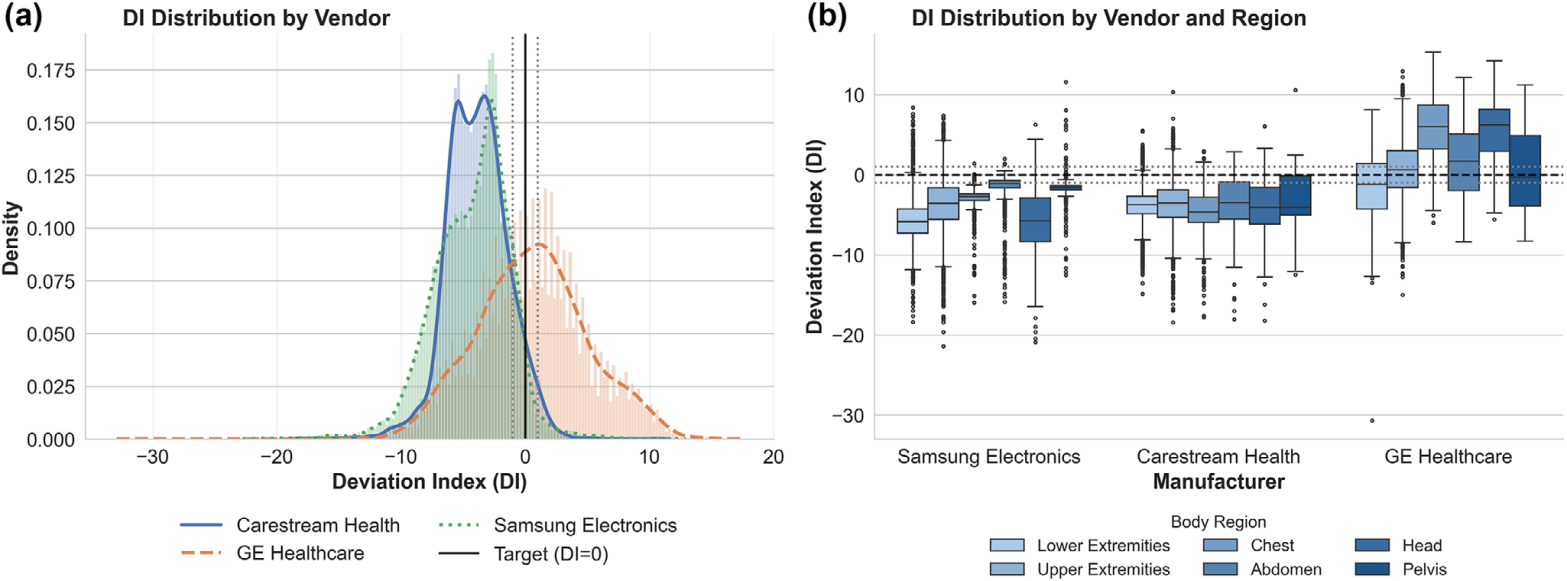
Vendor‐level deviation‐index (DI) distributions and boxplots (*N* = 38 916): Panel A overlays full‐cohort vendor‐specific DI histograms with kernel density estimates, highlighting how each platform is shifted relative to DI = 0 and the TG‐116 optimal band. The apparent Carestream double peak reflects the mixture of body region and projection groups within the vendor‐level display. Panel B shows DI boxplots by manufacturer and body region, underscoring the cross‐vendor differences that motivate the vendor‐region‐projection TEI‐setting review.

Across 38 916 examinations, the baseline DI distribution was strongly shifted toward negative values ($\bar{DI}\approx -3.1$, SD 3.7), and only 11.3% of examinations fell within |DI| ≤ 1. Table 1 quantifies the baseline group‐level TEI‐setting problem by vendor, Table 2 places that problem alongside standards‐based protocol adherence, and Figure 1 shows that the same bias is directly visible in the observed DI distribution.

**TABLE 1:** Baseline DI and TEI‐setting status by vendor.

Manufacturer	Total groups	$\bar{DI}$ range	Groups flagged (%)
Carestream Health	23	−4.82 to 0.73	95.7% (22/23)
GE Healthcare	16	−1.78 to 7.68	75.0% (12/16)
Samsung Electronics	6	−6.00 to −1.67	100.0% (6/6)

**TABLE 2:** Protocol adherence metrics and parallel TEI‐setting summaries. CAI, composite adherence index; SID, source‐to‐image distance; AEC, automatic exposure control; TEI, target exposure index. Percentages are examination‐level unless the row is explicitly labelled group‐level; N/A indicates a non‐applicable cell for a summary or group‐level metric.

Parameter	Standard	Tolerance	Out‐of‐spec N	Out‐of‐spec %	Composite score
SID	Region‐specific (100/180 cm)	±2%	19 069	49.0	0.098
kVp	Region/age‐specific	±5%	2696	6.9	0.014
Exposure (mAs)	Region/age‐specific	±10%	7609	19.6	0.039
Grid	Mandatory for adult torso and spine	Binary	3735	9.6	0.019
AEC	Recommended for adult torso	Binary	1057	2.7	0.005
TEI deviation (exam‐level)	Examination‐level |DI|	±1.0	34 504	88.7	N/A
TEI setting (group‐level)	Group $|\bar{DI}|$	±1.0	40	88.9	N/A
Total CAI	0 (perfect adherence)	N/A	N/A	17.6	0.176

Projection‐aware analysis refers to the evaluation of DI within vendor‐body‐region‐projection groups rather than only at the manufacturer or body‐region level. Table 1 shows that fixed DI offsets are pervasive across all vendors. The Samsung, Carestream, and GE systems showed group‐level flags of 100.0% (6/6), 95.7% (22/23), and 75.0% (12/16), respectively. Table S1 provides a complete vendor‐region‐projection breakdown for all 45 groups.

The $\bar{DI}$ ranges also differed by vendor, from −6.00 to −1.67 for Samsung, −4.82 to 0.73 for Carestream, and −1.78 to 7.68 for GE, respectively. The Samsung groups were uniformly negatively shifted, Carestream showed a dominant negative shift with one near‐centered group, and GE included both under‐ and over‐target groups. Therefore, the GE spread should not be interpreted as a single vendor‐wide target problem; it reflects heterogeneous group behavior.

For the five technical domains, the mean CAI contribution was calculated as the out‐of‐spec proportion divided by five. The percentages and CAI for SID, kVp, mAs, grid use, and AEC were computed for all 38 916 examinations, and TEI‐setting review was computed for 45 vendor‐region‐projection groups.

Among the protocol‐related factors, SID non‐compliance was the most frequent deviation (49.0%), followed by exposure (19.6%), grid use (9.6%), kVp (6.9%), and AEC (2.7%). When translated into the CAI scale, these corresponded to a mean CAI score of 0.176. In contrast, 88.7% of the examinations had |DI| > 1.0, and 88.9% of the vendor‐region‐projection groups exceeded the $|\bar{DI}|$ threshold of 1.0. Table 2 shows that protocol variation was present and should be reviewed; however, the dominant full‐cohort DI offset was not explained by protocol non‐compliance alone.

### 3.2 ML‐based variance discovery

The prespecified gradient boosting model achieved held‐out accuracy compatible with QA triage (RMSE 1.7; MAE 1.2 on the 20% evaluation subset). Because the ML analysis was secondary to the primary DI/TEI‐setting evaluation, comparative model performance results were not retained as a main‐text table.

**FIGURE 2:**
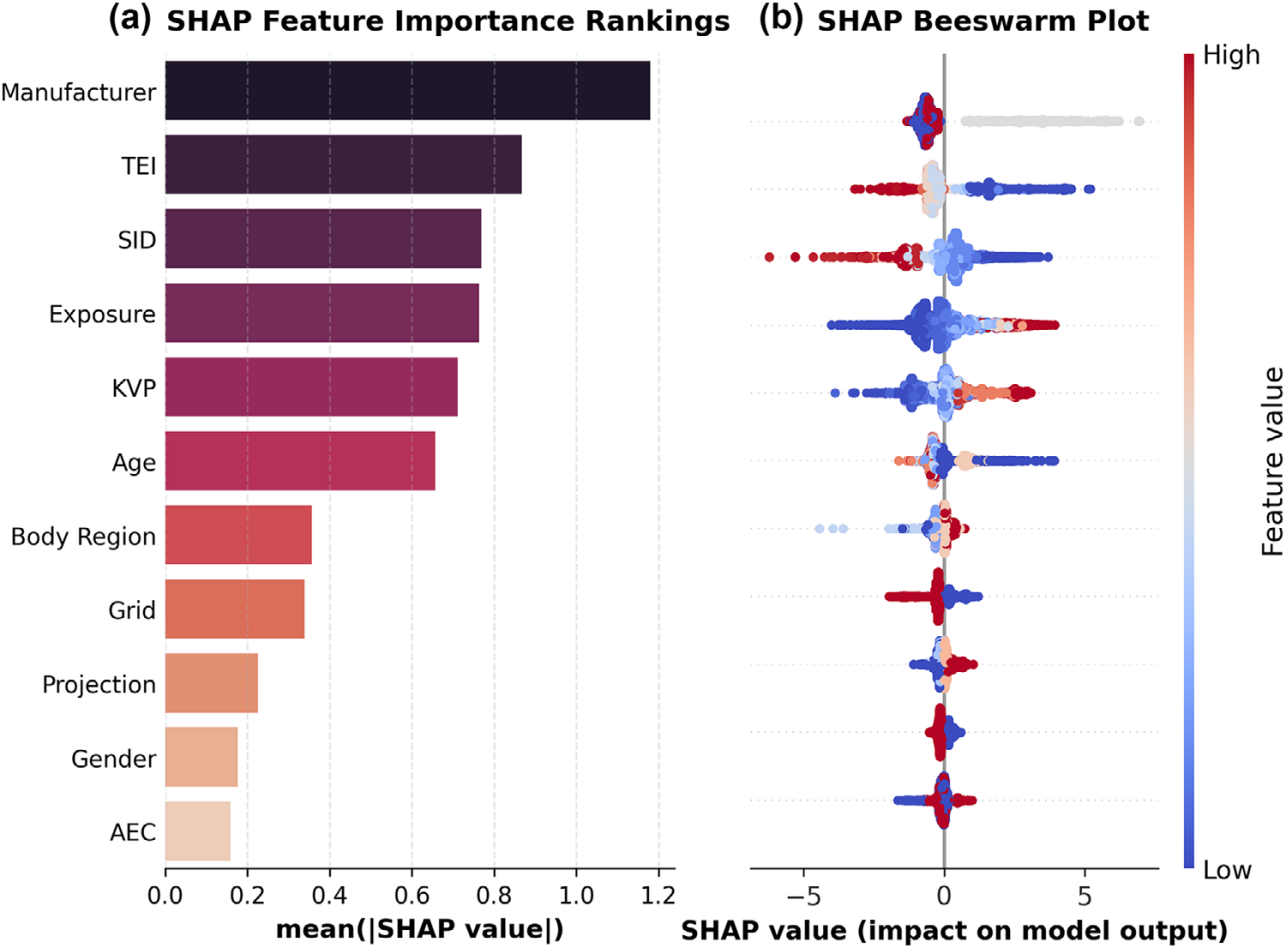
ML‐based variance discovery using SHAP: Panel A ranks the top predictors from the gradient boosting model based on the mean absolute SHAP values computed from 2000 randomly sampled held‐out examinations. Panel B presents a SHAP beeswarm plot, in which each dot represents an examination, the x‐axis position indicates the contribution to the model‐predicted DI, and the color denotes the low‐to‐high feature value.

Figure 2 shows that the configured TEI, manufacturer, SID, exposure, and kVp were high‐contribution predictors in the gradient boosting model by the mean absolute SHAP value. These rankings describe the model's reliance on metadata patterns when predicting DI. They were reported as predictive attribution only and did not prove that TEI caused the DI distribution or that changing TEI alone would optimize image quality or patient dose.

### 3.3 VR under TEI re‐centering simulation

When the DI was numerically re‐centered to zero within each vendor‐region‐projection group, the overall variance decreased by 42.9%, from 13.5 to 7.7. VR quantifies the fraction of the total DI variance removable under the counterfactual elimination of fixed group‐mean offsets; it is complementary to the between‐vendor ANOVA reported below for the kVp‐band analyses. Tables 3 and 4 quantify the baseline and counterfactual performances.

**TABLE 3:** Baseline clinical performance metrics by vendor. DI = deviation index; percentages were calculated within the listed cohort or vendor column.

Metric	Overall cohort	Samsung Electronics	Carestream Health	GE Healthcare
N	38 916	13 794	17 099	8023
$\bar{DI}$	−3.076	−4.252	−3.841	0.579
σ(DI)	3.670	3.019	2.451	4.457
$\sigma^{2}\left(DI\right)$	13.467	9.116	6.008	19.862
|DI| ≤ 1 (%)	11.337	9.149	9.638	18.721
|DI| > 3 (%)	60.335	62.440	64.249	48.373
Beyond ±2σ (%)	9.921	27.113	34.528	4.936

**TABLE 4:** Counterfactual performance after TEI re‐centering, with the group $\bar{DI}$ shifted to 0. DI: Deviation index; SD: Standard deviation; VR: Variance reduction. Percentages were calculated within the listed cohort or vendor columns.

Metric	Overall cohort	Samsung electronics	Carestream health	GE healthcare
σ(DI) after re‐centering	2.772	2.697	2.350	3.606
$\sigma^{2}\left(DI\right)$ after re‐centering	7.685	7.276	5.520	13.006
|DI| ≤ 1 (%)	34.261	42.475	34.154	20.366
|DI| > 3 (%)	23.134	20.357	15.831	43.475
Beyond ±2σ (%)	5.252	5.437	5.141	3.976
VR (%)	42.932	20.186	8.117	34.517

**FIGURE 3:**
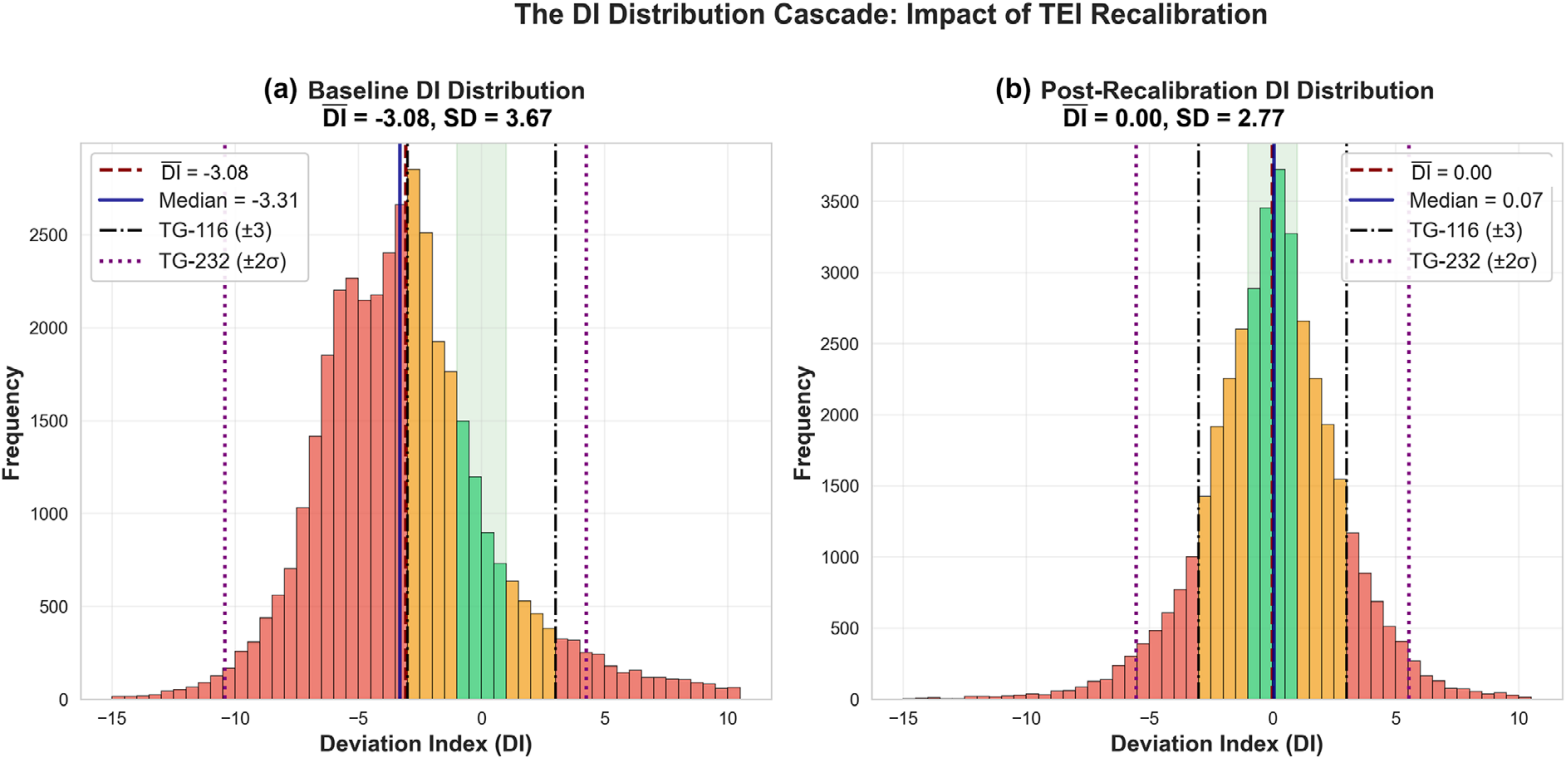
Full‐cohort DI distribution cascade with TG‐116/TG‐232 overlays: Side‐by‐side histograms comparing the baseline DI distribution with the post‐TEI‐centered counterfactual. The TG‐116 severity thresholds (± 1, ± 3) and TG‐232‐style ±2σ control limits are annotated to show how group‐level re‐centering removes fixed offsets while preserving within‐group spread. The histogram bars show the examination distributions before and after counterfactual recentring.

Figure 3 visualizes the shift in the full DI distribution from a biased monitoring signal to a more interpretable control distribution. Under this counterfactual state, the proportion of examinations within |DI| ≤ 1 rose from 11.3% to 34.3%, the proportion with |DI| > 3 fell from 60.3% to 23.1%, and the proportion beyond ± 2σ declined from 9.9% to 5.3%. Observations beyond the ± 2σ control limits may still reflect patient habitus, positioning, protocol selection, detector response, AEC behavior, or unusual clinical circumstances; re‐centering does not remove those sources of within‐group variation.

### 3.4 Robustness to operator, beam‐energy, and habitus effects

Sensitivity analyses were performed to test whether the observed DI variance could be explained by operator technique, beam energy, or patient habitus proxy measures rather than fixed group‐level offsets. Table 5 summarizes the AEC‐only restriction, beam energy subsets, VR estimates, and habitus correlations across all sensitivity subsets. Because the AEC mode decomposition required multiple cell‐level and inferential summaries, the full tabulation was moved to Table S2, while the main numerical findings are reported directly below.

**TABLE 5:** Overview of sensitivity analysis results, including baseline $\bar{DI}$, SD, baseline variance, re‐centered variance, variance reduction (VR), habitus correlation, and habitus $R^{2}$ values. DI: Deviation index; AEC: Automatic exposure control; kVp: Tube potential.

Subset	N	$\bar{DI}$	σ(DI)	Baseline variance	Re‐centered variance	VR (%)	Habitus corr. (r)	Habitus $R^{2}$
Full cohort	38 916	−3.076	3.670	13.467	7.685	42.932	0.248	0.062
AEC only	21 637	−3.443	2.548	6.490	4.832	25.552	0.346	0.120
kVp ≤ 70	24 703	−3.456	3.530	12.463	8.555	31.362	0.154	0.024
kVp 71–90	7072	−2.298	3.689	13.609	8.194	39.791	0.465	0.216
kVp 91–110	4068	−4.341	1.998	3.993	3.337	16.447	0.220	0.048
kVp > 110	3071	−0.128	4.518	20.416	3.456	83.074	0.055	0.003

To address the vendor‐correlated structure of AEC uptake (99.9% Carestream, 24.5% Samsung, 14.6% GE AEC rates), the AEC‐only subset (*N* = 21 637) was interpreted alongside an explicit vendor × AEC‐mode decomposition provided in Table S2 rather than as a vendor‐neutral control. Within cells, the Carestream AEC $\bar{DI}$ was −3.8 (SD2.5); the Samsung AEC $\bar{DI}$ was −2.5 (SD1.5) versus Samsung MANUAL −4.8 (SD3.2); and the GE AEC $\bar{DI}$ was −0.5 (SD3.5) versus GE MANUAL +0.8 (SD4.6). Within‐vendor Welch t‐tests indicated that AEC‐mode effects on DI bias were vendor‐specific rather than uniformly protective: Samsung AEC exams were 2.4 DI units less biased than Samsung manual exams (Welch *t* = 59.8, *p* < 0.001, bootstrap 95% CI 2.3 to 2.5), whereas GE AEC exams were 1.2 DI units more biased than GE manual exams (Welch *t* = 10.6, *p* < 0.001, bootstrap 95% CI −1.4 to −1.0). A two‐way ANOVA on the balanced Samsung and GE subset (*N* = 21 817) decomposed DI variance into vendor ($\eta_{p}^{2}$ = 0.314), AEC‐mode ($\eta_{p}^{2}$ = 0.024), and their interaction ($\eta_{p}^{2}$ = 0.034); the full model $\eta^{2}$ was 0.333, indicating that the vendor explained approximately 13 times the DI variance attributed to the AEC mode. Within the vendor, the AEC mode was a minor contributor to the DI offset, whereas the vendor‐fixed effects dominated. Vendor‐specific DI offsets also persisted within every kVp band (Welch's ANOVA, all *p* < 0.001, with $\eta^{2}$ = 0.237 for ≤ 70 kVp, 0.223 for 71–90 kVp, 0.079 for 91–110 kVp, and 0.813 for > 110 kVp). The 91–110 kVp band showed the smallest vendor effect, reflecting its composition of 99.3% Carestream. The corresponding vendor‐region‐projection VR estimates ranged from 16.4% in the 91–110 kVp band to 83.1% in the > 110 kVp band, and in the AEC‐only subset, the same vendor‐region‐projection re‐centering removed 25.6% of the DI variance. These findings are consistent with fixed TEI‐setting offsets across beam energy ranges and show that the AEC imbalance in the AEC‐only subset reflects the Carestream composition rather than a vendor‐neutral exposure‐mode control.

To test whether patient size could account for the observed DI behavior, we used log10(1 + mAs) as a proxy for patient habitus. In the full cohort, DI showed a weak positive correlation with the habitus proxy (r=0.248; $R^{2}=0.062$), explaining approximately 6% of the variance. The association was stronger in AEC‐only examinations (r=0.346; $R^{2}=0.120$), where mAs more directly reflected patient attenuation. Across the kVp bands, the habitus correlations ranged from 0.055 in the > 110 kVp band ($R^{2}=0.003$) to 0.465 in the 71–90 kVp band ($R^{2}=0.216$). In no subset did the habitus proxy explain more than 22% of the DI variance, indicating that patient size contributed to DI variability but was not the dominant source of the observed group‐level offsets.

Taken together, these sensitivity results indicate that patient habitus and beam energy contributed to the DI variability but did not account for the dominant group‐level patterns observed in the main analysis. Even within the technically constrained AEC‐only subset, recentering removed a substantial portion of the DI variance. The persistence of vendor‐specific offsets across all kVp bands, combined with modest habitus correlations, supports the interpretation that fixed TEI‐setting offsets were the largest removable source of DI variance under the counterfactual model.

## 4 DISCUSSION

### 4.1 Evidence for fixed DI offsets after protocol screening

This study showed that DI behavior in a multi‐vendor DR environment contained large fixed group‐level offsets that were not explained by protocol noncompliance alone. The baseline evidence package makes this sequence explicit in the following way. Table 1 quantifies the vendor‐region‐projection group problems. Table 2 shows that standards‐based protocol non‐compliance was present. Because CAI is a unitless 0‐to‐1 proportion whereas DI and DI variance use different scales, their numerical magnitudes were not compared; interpretation instead relied on the pattern and co‐occurrence of protocol deviations and group‐level DI offsets. Figure 1 shows that these shifts are directly visible in the observed distribution of the DI. Across nearly 39 000 examinations, the DI distributions were systematically shifted away from zero, and 40 of the 45 vendor‐region‐projection groups (88.9%) exceeded a prespecified operational TEI‐review threshold of $|\bar{DI}|$ > 1.0, informed by the TG‐116 examination‐level DI action band. The secondary ML triage step provided complementary, non‐causal support, as shown in Figure 2, where the predictive model relied heavily on the configured TEI and manufacturer metadata. ML analysis was not retained as a primary deliverable but as a data‐driven cross‐check on the standards‐based CAI analysis. CAI evaluates protocol adherence against externally published rules, whereas SHAP evaluates which metadata patterns a predictive model relies on when estimating DI from the data itself. Because protocol‐adherence screening did not explain the group‐level DI offsets and SHAP ranked the configured TEI as a high‐contribution predictor, the combined evidence strengthens the rationale for a structured TEI‐setting review; neither analysis alone establishes causality. ML‐based triage may also be adaptable to departments with sufficient DICOM metadata but have not yet implemented the full CAI rule set.

The same interpretation was supported when the likely confounders were constrained. Within‐vendor AEC‐mode contrasts and a two‐way ANOVA on the balanced Samsung and GE subset decomposed DI variance into vendor ($\eta_{p}^{2}$ = 0.314), AEC‐mode ($\eta_{p}^{2}$ = 0.024), and their interaction ($\eta_{p}^{2}$ = 0.034); vendor explained approximately 13 times the DI variance attributed to the AEC mode, indicating that the exposure mode alone was a minor contributor to the DI offset once the vendor was accounted for. Although the AEC‐only subset was Carestream‐dominated rather than vendor‐neutral, vendor‐region‐projection re‐centering still removed 25.6% of the DI variance within it. Habitus correlations remained modest in all subsets (maximum $R^{2}=0.216$), and vendor offsets persisted across all kVp bands (Welch's ANOVA, *p* < 0.001, F‐statistics 43.7 to 3328.3, effect sizes ranging from $\eta^{2}$ = 0.079 to 0.813). Taken together, these findings support the TEI‐setting review as a practical QA target, while leaving room for AEC, detector, protocol, and clinical image‐quality factors to be evaluated before implementation.

### 4.2 Re‐centering DI before σ‐based monitoring

The recentering results clarified the relationship between TG‐116 and TG‐232. Tables 3 and 4 quantify the extent to which the observed DI spread can be removed by vendor‐region‐projection re‐centering, and Figure 3 shows why this correction changes the interpretability of the DI. TG‐116 introduced the algebraic definition of the DI and proposed fixed interpretive bands; however, these bands are meaningful only when the configured TEI is appropriate for the protocol. TG‐232 recognized that clinical distributions vary and recommended local SD‐based action limits once the $\bar{DI}$ is centered.^10^ The proposed framework bridges these two perspectives.

In practical terms, TG‐116‐style thresholds are useful as a screening tool for persistent DI offset, whereas TG‐232‐style σ‐based limits are meaningful only after the distribution has been centered around an appropriate target. In the uncorrected state, fixed thresholds classify most examinations as noncompliant because the distribution itself is biased. After centering, only a smaller tail remains outside the local σ‐based limits, which is consistent with a more stable statistical process. In this framework, the TEI‐setting review functions as an enabling step for TG‐232‐style governance.

### 4.3 Practical TEI‐setting workflow and safeguards

**FIGURE 4:**
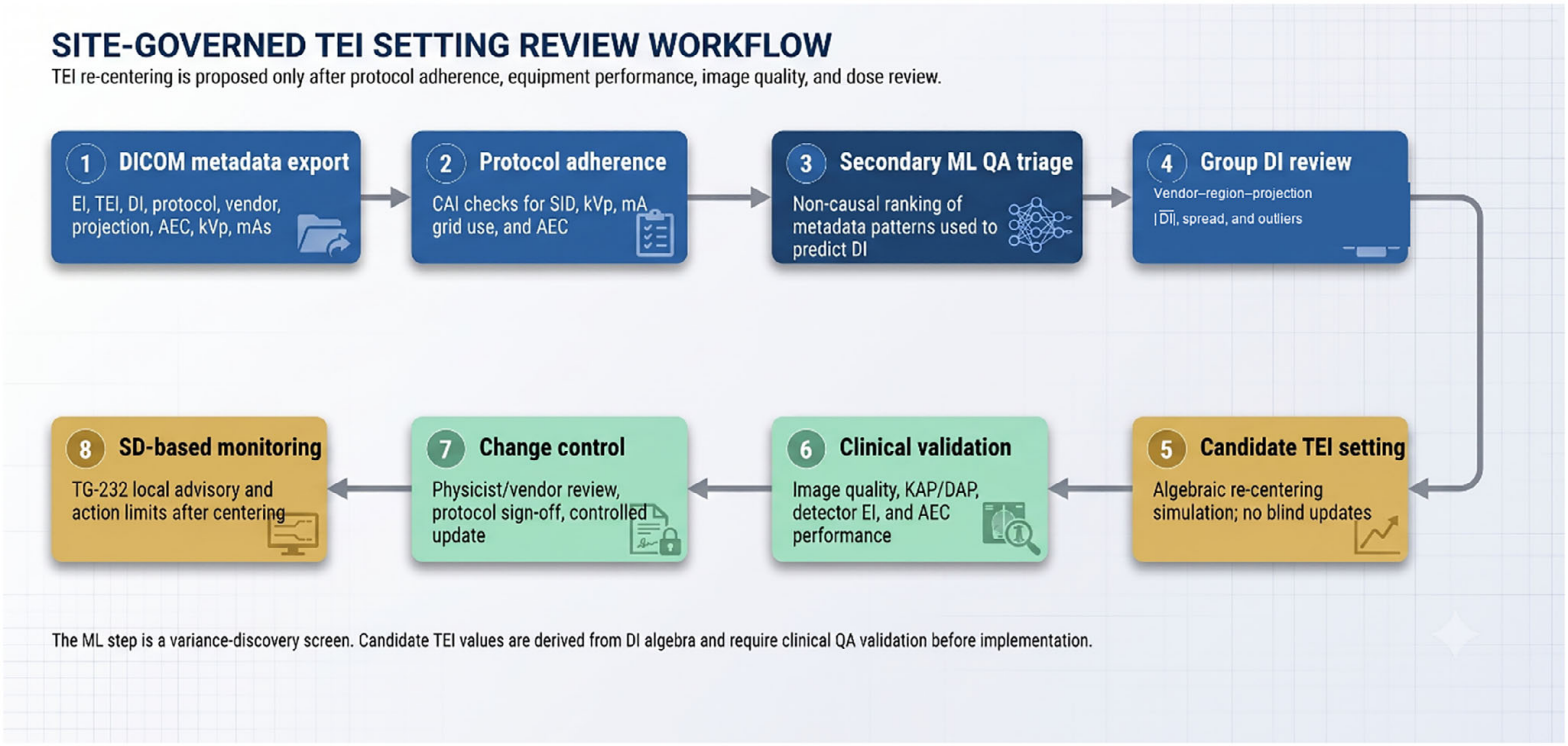
Site‐governed TEI‐setting review workflow schematic: The workflow summarizes DICOM‐derived metric ingestion, standards‐aware protocol checks using CAI (composite adherence index), non‐causal ML/SHAP variance discovery, vendor‐region‐projection DI review, candidate TEI recentering simulation, image quality and dose review including KAP/DAP where available, controlled TEI update, and SD‐based monitoring.

For a clinical department, the framework can be translated into a practical workflow, as shown in Figure 4.

1. Baseline audit: Computation of DI distributions and TEI‐implied summaries by vendor, body region, and projection; flag groups with $|\bar{DI}|$ > 1.0.
2. Convergent variance discovery: Use gradient boosting with SHAP only to rank the metadata patterns used by the model to predict DI; interpret this as non‐causal variance discovery.
3. TEI proposal generation: Deriving candidate TEI values for flagged groups and producing site‐specific TEI update proposals consistent with local protocol intent.
4. Clinical validation and controlled implementation: Review representative images, KAP/DAP or available patient dose indicators, EI/detector performance, AEC calibration, and chamber behavior, then submit approved TEI changes through formal change control and re‐measure DI distributions after deployment.
5. SD‐based monitoring: Once the $\bar{DI}$ has been centered, local ± 1σ and ± 2σ bands can be established as advisory and action thresholds to focus QA effort on persistent outliers and drifts.

This workflow is intended to convert routine DICOM exports into a vendor‐neutral TEI review process and provides the clinical site with a structured role in TEI governance while preserving vendor involvement for detector EI and AEC calibration. It should not be implemented blindly; if the underlying clinical data reflect systematically poor technique or unacceptable image quality, re‐centering the DI would simply normalize an undesirable exposure practice.

### 4.4 Complementary role of phantom‐based TEI methods

The clinical data workflow proposed here is complementary to the hybrid method of Hoerner et al.,^26^ which combines automatic exposures of flat‐field phantoms with filtered clinical EI data to derive vendor‐ and protocol‐specific scaling factors for target exposure indices. The phantom component reduces patient habitus, positioning, and protocol‐mix confounding and is well suited to commissioning, AEC verification, or focused equipment investigation, whereas the clinical‐data component connects those controlled measurements to routine practice.

The present method addresses a different operational question: how configured TEI values behave across the actual mixture of vendors, protocols, projections, patient sizes, and technologist practices encountered in routine care. Therefore, the clinical data method should be interpreted as a surveillance and proposal‐generation tool. Phantom measurements, AEC testing, task‐based image quality assessment, and patient dose review remain important when deciding whether a proposed TEI value is clinically appropriate.

### 4.5 Sensitivity analysis implications

The robustness analysis presented in Table 5 and in Table S2 supports the central interpretation of the proposed framework. In the full cohort, the habitus correlation was weak (r=0.248), and even in the AEC‐only subset, the correlation remained modest (r=0.346). Across all kVp bands, the habitus proxy explained no more than 22% of the DI variance, while re‐centering removed fixed offsets in every subset. These results suggest that patient habitus should be understood as a contributing factor but an insufficient explanation for the observed DI offset. The AEC‐only subset was compositionally biased toward Carestream (99.9% AEC usage), which limited its generalizability as a vendor‐neutral control; nevertheless, vendor‐region‐projection re‐centering still removed 25.6% of the DI variance within it. The balanced two‐vendor subset confirmed the dominance of vendor over exposure mode: a two‐way ANOVA identified vendor $\eta_{p}^{2}$ = 0.314, AEC‐mode $\eta_{p}^{2}$ = 0.024, and interaction $\eta_{p}^{2}$ = 0.034, implying that the AEC mode effects on DI bias are vendor‐specific rather than uniformly protective; within‐vendor Welch t‐tests showed that Samsung AEC exams were 2.4 DI units less biased than manual exams, whereas GE AEC exams were 1.2 DI units more biased. The > 110 kVp band, dominated by chest imaging, showed the highest vendor effect ($\eta^{2}$ = 0.813) and VR (83.1%), reflecting the extreme vendor offsets in this range and suggesting that high‐kVp protocols may be priority targets for TEI‐setting review. These findings reinforce the workflow requirements for AEC calibration verification, detector EI testing, and image quality review before any TEI update is implemented.

### 4.6 Limitations and future directions

This study had several limitations. First, the TEI re‐centering analysis was counterfactual; candidate TEI updates were simulated mathematically rather than implemented prospectively in clinical systems. This isolates the expected effect of removing the fixed group‐level DI offset but does not capture how staff may respond behaviorally to updated DI feedback targets. Second, the study was conducted at a single center; therefore, the specific TEI values derived here should not be directly transferred to other institutions without local validation. Third, the available DICOM extracts did not include clinical image quality scores, observer assessments, KAP/DAP, entrance air kerma, or BMI for all examinations. These measures are essential before TEI updates are implemented because recentering a DI distribution derived from a poor technique could support underexposure or dose creep rather than appropriate optimization. Fourth, the body‐region groupings used for analysis (e.g., lower extremities) were exploratory and defined by the available DICOM body‐part fields; final TEI decisions should be made at the vendor‐projection level for each specific protocol unless the local protocol design intentionally shares a target across projections. Fifth, AEC uptake was strongly vendor‐correlated (99.9% Carestream, 14.6% GE, 24.5% Samsung); therefore, the AEC‐only sensitivity analysis could not fully disentangle vendor‐fixed effects from exposure‐mode effects. Within‐vendor AEC‐versus‐MANUAL contrasts and the two‐way ANOVA on the Samsung‐GE subset (Section 3.4) partially address this confound but cannot eliminate it for Carestream.

However, the framework is portable. Departments with sufficiently complete routine DICOM metadata can reproduce the same workflow and generate a local TEI‐setting profile that matches their own detectors, protocols, and clinical preferences. Implementation can be scaled to local resources. Large centers may automate DICOM aggregation across vendors, whereas smaller or resource‐limited departments can begin with periodic exports for a few high‐volume protocols and omit the optional ML/SHAP layer. At minimum, reliable EI, TEI, DI, vendor, projection, and technique metadata; adequate group counts; medical‐physicist oversight; image‐quality and dose review; and formal vendor change control are required. Where metadata are sparse or unreliable, phantom measurements and manual audits should precede any TEI proposal, and all candidate values remain site‐specific. Future studies should include the prospective implementation of revised TEI tables, formal AEC and detector verification, image‐quality assessment with human observers or task‐based methods, incorporation of patient dose metrics such as KAP/DAP, and iterative optimization of exposure factors once the DI is stabilized.^7^, ^8^ A stabilized DI signal could later support AI‐assisted QC, but such extensions should follow and not replace TEI review and equipment quality assurance.^11^

## 5 CONCLUSION

This retrospective multi‐vendor study evaluated a TEI‐setting review framework aligned with the transition from TG‐116 to TG‐232 guidelines. By treating TEI review as a data‐driven clinical governance task rather than a passive vendor default, the framework enables departments to:

- Identify vendor‐region‐projection groups in which fixed DI offset, rather than operator performance alone, dominates DI variability
- Derive candidate TEI targets from routine clinical data to generate defensible vendor‐neutral TEI update proposals
- Quantify the expected impact of TEI re‐centering before implementation through counterfactual variance‐reduction analysis
- Create the centered baseline required for meaningful σ‐based DI monitoring under TG‐232

In multi‐vendor radiography environments, this approach provides a practical framework for moving from biased DI reporting to stable process control. Once protocol adherence, image quality, dose indicators, detector EI calibration, and AEC performance have been reviewed and the $\bar{DI}$ is centered, the DI can be interpreted as intended: a focused indicator of genuine outliers rather than a noisy artifact of poorly aligned TEI settings.

## AUTHOR CONTRIBUTIONS

**Ezzat AbuAzzah**: Conceptualization; methodology; resources; software; data curation; investigation; formal analysis; writing—original draft. **Faisal Alrehily**: Review and editing. **Moawia Gameraddin**: Review and editing. **Sultan Alshoabi**: Review and editing. **Walaa Alsharif**: Review and editing. **Kamal Dahan Alsultan**: Review and editing. **Abdullah Alshamrani**: Review and editing. **Khaled Al‐Radadi**: Resources; data curation. **Ramy Badawy**: Resources; data curation. **Samer Al‐Jabri**: Formal analysis. **Turki Al‐Harbi**: Formal analysis. **Nawaf Al‐Hazme**: Formal analysis. All authors have read and approved the final manuscript.

## CONFLICT OF INTEREST STATEMENT

The authors declare no conflicts of interest.

## ETHICAL APPROVAL

This study was conducted in accordance with the principles of the Declaration of Helsinki. Institutional Review Board approval was obtained for the retrospective analysis of anonymized DICOM metadata (approval no. 25‐003). Although this study utilized patient‐derived data, it did not involve direct interactions with human participants or animals. All data were fully anonymized prior to analysis to ensure patient confidentiality and privacy.

## GENERATIVE AI STATEMENT

Claude and Gemini were used to assist with data analysis‐related work, including analytical exploration and interpretation support during manuscript development. SciSpace was used to support literature discovery and screenings. PaperPal was used for language editing and refinement. The revised workflow schematic was generated programmatically by the authors of this study. AI tools were not used as autonomous authors or agents in this study. All AI‐assisted outputs were reviewed, verified, and revised by the authors. The authors retain full responsibility for the study design, analyses, interpretation, figures, and final manuscript content. AI tools were not mentioned as authors.

## Supporting information




**Supporting Information**: acm270820‐sup‐0001‐TableS1.rtf

**Supporting Information**: acm270820‐sup‐0002‐TableS2.rtf

## Data Availability

De‐identified aggregate outputs and analysis code are available from the corresponding author upon reasonable request, subject to institutional and ethical approvals. Examination‐level clinical metadata cannot be shared publicly because they are derived from a local clinical PACS export.
