# Supplementary material for: Bridging TG‐116 and TG‐232: A retrospective multi‐vendor evaluation of deviation index re‐centering and process control in digital radiography
Source: J Appl Clin Med Phys. 2026 Sep 23;27(10):e70820. doi: 10.1002/acm2.70820 (PMC13601757; doi:10.1002/acm2.70820)
Supplement: Supplementary file 1 [file ACM2-27-e70820-s001.rtf]

Supplemental Table S2. AEC-mode sensitivity decomposition
This supplemental table reports the AEC-mode decomposition used to support the Results and Discussion. It is provided as supplementary material because the complete decomposition requires cell-level, within-vendor, and factorial ANOVA summaries that are too detailed for a compact main-text table. The main manuscript reports the key numerical findings in prose.
Panel A. Vendor × AEC-mode cell counts and group-level DI statistics.
Vendor	AEC mode	N	Mean DI	SD	
Carestream Health	AEC	17,085	-3.8	2.5	
Samsung Electronics	AEC	3,379	-2.5	1.5	
Samsung Electronics	MANUAL	10,415	-4.8	3.2	
GE Healthcare	AEC	1,173	-0.5	3.5	
GE Healthcare	MANUAL	6,850	0.8	4.6	

Panel B. Within-vendor AEC-versus-MANUAL contrasts for vendors with both exposure modes represented.
Vendor	AEC mean DI	MANUAL mean DI	Delta (AEC -
MANUAL)	Welch t	p-value	Bootstrap
95% CI of Delta	
Samsung
Electronics	-2.5	-4.8	2.4	59.8	<0.001	2.3 to 2.5	
GE Healthcare	-0.5	0.8	-1.2	10.6	<0.001	-1.4 to -1.0	

Panel C. Two-way ANOVA decomposition of DI variance in the balanced Samsung and GE subset.
Effect (two-way ANOVA on Samsung
+ GE subset, N = 21,817)	Partial eta-squared	Total eta-squared (model R-
squared)	
Vendor (Samsung vs GE)	0.314	0.333	
AEC mode	0.024	0.333	
Vendor × AEC-mode interaction	0.034	0.333	

Abbreviations: AEC, automatic exposure control; DI, deviation index; CI, confidence interval.
Carestream Health was excluded from the two-way ANOVA because it contributed essentially no MANUAL examinations and therefore could not support a factorial AEC-mode comparison.
