# Supplementary material for: Bridging TG‐116 and TG‐232: A retrospective multi‐vendor evaluation of deviation index re‐centering and process control in digital radiography
Source: J Appl Clin Med Phys. 2026 Sep 23;27(10):e70820. doi: 10.1002/acm2.70820 (PMC13601757; doi:10.1002/acm2.70820)
Supplement: Supplementary file 2 [file ACM2-27-e70820-s002.rtf]

Supplemental Table S1. Vendor–region–projection breakdown
Manufacturer	Body region	Projection	N	Mean DI	SD DI	Median EI	Median TEI	|DI| > 3 (%)	AEC (%)	TEI factor	|Mean DI| > 1	
Carestream Health	Abdomen	AP	1943	-3.187	2.575	150.420	226.220	49.820	100.000	0.480	Yes	
Carestream Health	Abdomen	LAT	179	-4.495	2.448	133.160	438.000	78.212	100.000	0.355	Yes	
Carestream Health	Abdomen	Other	6	-3.253	2.514	150.925	332.110	66.667	100.000	0.473	Yes	
Carestream Health	Abdomen	PA	8	-3.193	2.890	150.010	226.220	62.500	100.000	0.479	Yes	
Carestream Health	Chest	AP	678	-3.857	2.472	129.940	226.220	59.440	100.000	0.411	Yes	
Carestream Health	Chest	LAT	62	-3.086	3.001	160.960	226.220	46.774	100.000	0.491	Yes	
Carestream Health	Chest	PA	3699	-4.544	1.823	114.880	438.000	73.371	100.000	0.351	Yes	
Carestream Health	Head	AP	164	-3.932	2.827	142.945	438.000	60.976	100.000	0.404	Yes	
Carestream Health	Head	LAT	191	-4.822	3.836	122.490	438.000	67.016	100.000	0.329	Yes	
Carestream Health	Head	Other	10	-4.581	5.385	178.390	226.220	60.000	100.000	0.348	Yes	
Carestream Health	Head	PA	37	-1.031	2.701	222.440	438.000	40.541	100.000	0.789	Yes	
Carestream Health	Lower Extremities	AP	3660	-3.766	2.361	157.120	438.000	67.404	100.000	0.420	Yes	
Carestream Health	Lower Extremities	LAT	1762	-3.882	2.114	154.880	438.000	73.269	100.000	0.409	Yes	
Carestream Health	Lower Extremities	Other	7	-1.044	3.610	182.480	438.000	42.857	100.000	0.786	Yes	
Carestream Health	Lower Extremities	PA	4	-4.650	3.344	123.650	226.220	75.000	100.000	0.343	Yes	
Carestream Health	Pelvis	AP	385	-1.178	2.407	211.700	226.220	21.299	100.000	0.762	Yes	
Carestream Health	Pelvis	LAT	336	-4.769	1.556	140.460	438.000	93.452	100.000	0.333	Yes	
Carestream Health	Pelvis	Other	4	-4.520	2.638	128.360	438.000	75.000	100.000	0.353	Yes	
Carestream Health	Pelvis	PA	2	0.730	0.311	268.160	226.220	0.000	100.000	1.183	No	
Carestream Health	Upper Extremities	AP	1245	-4.552	2.921	130.940	438.000	73.735	99.598	0.351	Yes	
Carestream Health	Upper Extremities	LAT	1101	-3.844	3.000	141.500	438.000	61.762	99.364	0.413	Yes	
Carestream Health	Upper Extremities	Other	3	-2.757	3.037	125.050	226.220	66.667	100.000	0.530	Yes	
Carestream Health	Upper Extremities	PA	1613	-2.942	2.053	179.320	438.000	44.079	99.876	0.508	Yes	
GE Healthcare	Abdomen	AP	271	0.338	3.495	231.341	296.000	40.959	46.494	1.081	No	
GE Healthcare	Abdomen	LAT	236	3.156	4.371	835.914	292.400	74.153	21.610	2.068	Yes	
GE Healthcare	Chest	AP	143	3.560	2.233	243.779	105.020	65.734	88.112	2.270	Yes	
GE Healthcare	Chest	LAT	25	2.780	4.953	507.129	206.000	72.000	40.000	1.897	Yes	
GE Healthcare	Chest	PA	811	6.418	3.229	538.880	105.020	80.025	37.485	4.383	Yes	
GE Healthcare	Head	AP	77	2.987	3.494	476.664	182.830	61.039	33.766	1.989	Yes	
GE Healthcare	Head	LAT	106	7.681	3.246	562.825	91.000	89.623	15.094	5.863	Yes	


Supplemental Table S1. Vendor–region–projection breakdown (continued)
Manufacturer	Body region	Projection	N	Mean DI	SD DI	Median EI	Median TEI	|DI| > 3 (%)	AEC (%)	TEI factor	|Mean DI| > 1	
GE Healthcare	Head	PA	5	3.380	2.345	305.830	155.600	40.000	60.000	2.178	Yes	
GE Healthcare	Lower Extremities	AP	2977	-1.782	3.854	497.021	718.200	46.960	6.752	0.663	Yes	
GE Healthcare	Lower Extremities	LAT	367	0.756	2.736	815.469	718.200	25.341	0.000	1.190	No	
GE Healthcare	Lower Extremities	Other	26	2.127	1.939	707.784	416.900	26.923	0.000	1.632	Yes	
GE Healthcare	Pelvis	AP	176	0.415	4.813	319.236	363.000	64.205	33.523	1.100	No	
GE Healthcare	Pelvis	LAT	11	4.427	4.261	1503.594	332.595	81.818	9.091	2.772	Yes	
GE Healthcare	Upper Extremities	AP	1158	1.361	3.754	647.049	480.600	41.364	21.589	1.368	Yes	
GE Healthcare	Upper Extremities	LAT	150	2.197	2.558	973.713	653.050	27.333	0.000	1.659	Yes	
GE Healthcare	Upper Extremities	PA	1484	-0.060	3.305	749.189	658.600	37.062	0.000	0.986	No	
Samsung Electronics	Abdomen	Other	887	-1.717	2.228	388.000	500.000	11.725	98.422	0.673	Yes	
Samsung Electronics	Chest	Other	2235	-2.872	1.034	265.000	500.000	33.647	99.060	0.516	Yes	
Samsung Electronics	Head	Other	277	-5.996	4.347	130.000	500.000	75.090	0.000	0.251	Yes	
Samsung Electronics	Lower Extremities	Other	5328	-5.710	2.611	128.000	500.000	88.307	0.000	0.269	Yes	
Samsung Electronics	Pelvis	Other	363	-1.674	2.151	348.000	500.000	11.570	80.441	0.680	Yes	
Samsung Electronics	Upper Extremities	Other	4704	-3.831	3.273	214.000	500.000	59.566	0.000	0.414	Yes	

Each row summarizes a manufacturer, body-region, and projection group. The TEI factor is 10^(mean DI/10), the multiplicative factor implied by algebraic re-centering. Rows flagged as |mean DI| > 1 require clinical review before any TEI update is implemented.
